# Evolution of Meniscal Biomechanical Properties with Growth: An Experimental and Numerical Study

**DOI:** 10.3390/bioengineering8050070

**Published:** 2021-05-20

**Authors:** Marco Ferroni, Beatrice Belgio, Giuseppe M. Peretti, Alessia Di Giancamillo, Federica Boschetti

**Affiliations:** 1 Department of Chemistry, Materials and Chemical Engineering “Giulio Natta”, Politecnico di Milano, 20133 Milan, Italy; marco.ferroni@polimi.it (M.F.); beatrice.belgio@polimi.it (B.B.); 2 IRCCS Istituto Ortopedico Galeazzi, 20161 Milan, Italy; giuseppe.peretti@unimi.it; 3 Department of Biomedical Sciences for Health, Università degli Studi di Milano, 20133 Milan, Italy; 4 Department of Health, Animal Science and Food Safety, Università degli Studi di Milano, 20133 Milan, Italy; alessia.digiancamillo@unimi.it

**Keywords:** meniscus, growth, mechanical properties, swine

## Abstract

The menisci of the knee are complex fibro-cartilaginous tissues that play important roles in load bearing, shock absorption, joint lubrication, and stabilization. The objective of this study was to evaluate the interaction between the different meniscal tissue components (i.e., the solid matrix constituents and the fluid phase) and the mechanical response according to the developmental stage of the tissue. Menisci derived from partially and fully developed pigs were analyzed. We carried out biochemical analyses to quantify glycosaminoglycan (GAG) and DNA content according to the developmental stage. These values were related to tissue mechanical properties that were measured in vitro by performing compression and tension tests on meniscal specimens. Both compression and tension protocols consisted of multi-ramp stress–relaxation tests comprised of increasing strains followed by stress–relaxation to equilibrium. To better understand the mechanical response to different directions of mechanical stimulus and to relate it to the tissue structural composition and development, we performed numerical simulations that implemented different constitutive models (poro-elasticity, viscoelasticity, transversal isotropy, or combinations of the above) using the commercial software COMSOL Multiphysics. The numerical models also allowed us to determine several mechanical parameters that cannot be directly measured by experimental tests. The results of our investigation showed that the meniscus is a non-linear, anisotropic, non-homogeneous material: mechanical parameters increase with strain, depend on the direction of load, and vary among regions (anterior, central, and posterior). Preliminary numerical results showed the predominant role of the different tissue components depending on the mechanical stimulus. The outcomes of biochemical analyses related to mechanical properties confirmed the findings of the numerical models, suggesting a specific response of meniscal cells to the regional mechanical stimuli in the knee joint. During maturation, the increase in compressive moduli could be explained by cell differentiation from fibroblasts to metabolically active chondrocytes, as indicated by the found increase in GAG/DNA ratio. The changes of tensile mechanical response during development could be related to collagen II accumulation during growth. This study provides new information on the changes of tissue structural components during maturation and the relationship between tissue composition and mechanical response.

## 1. Introduction

The menisci are two semilunar fibro-cartilaginous structures medially and laterally interposed between the femoral condyles and the tibial plateau in the knee joint. They play key roles in load bearing, shock absorption, joint stability, congruity, lubrication, and nutrient distribution for articular cartilage [1,2,3,4,5]. The meniscus appears wedge-shaped in cross-section and slightly concave on the femoral surface. The tissue is mainly composed of water, which constitutes the 70–75% of the wet weight, and the remaining parts are 60–70% collagen, 1% proteoglycans, and 8–13% non-collagenous proteins such as elastin [6]. Though collagens are primarily type I (90%), type 2 collagen is also present [6,7]. Collagen fibers have a predominantly a circumferential orientation, with only some radially oriented fibers. Structural and biochemical changes occurring in the swine meniscus have demonstrated an increasing enrichment in cartilaginous components during their development [8].

Under normal physiologic loading, the meniscus experiences large tensile, shear, and compressive stress, which can lead to injury [2,9]. Unfortunately, the tissue has a poor healing potential, mainly due to its limited vascularization [10]. Recently, total meniscectomy has been abandoned because of its relationship with the development of osteoarthritis. Nowadays, the treatment goal is to remove the damaged meniscus and rebuild it or, if necessary, replace it by using tissue regeneration therapies. Many attempts have already been made, e.g., those by Ding et al. [11], Ionescu et al. [12], and Osawa et al. [13]. Detailed knowledge of meniscal composition, structure, and mechanical properties is required to develop effective tissue regenerative strategies. 

Previous studies have demonstrated that the biomechanical properties of the meniscus are site- and depth-specific [14,15,16,17,18,19]. For instance, Proctor et al. [14] evaluated the compressive and tensile properties of bovine menisci and their variation with anatomical location. Similarly, Danso et al. [16] reported that under compression, the mechanical properties of the human meniscus varied according to the site, i.e., anterior, middle and posterior site. In addition, in a study by Peretti et al. [18], femoral and tibial surfaces displayed different biomechanical responses, apart from the dependency on meniscus sites. Moreover, that the structure of meniscus fiber is composed of two orthogonal collagenous networks, radial and circumferential, has an impact on tissue mechanical properties [20,21]. Peloquin et al. [20] performed meniscal uniaxial tensile test along radial and circumferential directions, and they found that radially loaded specimens were less stiff than those loaded circumferentially. Norberg et al. [22] investigated the meniscal shear properties in relation to radial and circumferential direction, as well as to tissue composition. However, how the structural constituents contribute to the meniscal mechanical properties is not yet fully understood, probably due to the shortage of studies on meniscal mechanical properties. Moreover, little is known about changes related to growth and aging in the meniscus’ mechanical properties. Several studies have indicated that the meniscal extracellular matrix degenerates with aging, thus leading to changes in the compositional structure of the meniscus and non-physiological loading, which significantly affects overall joint health [23,24]. Kwok et al. [24] assessed the nanomechanical properties of young healthy, aged, and osteoarthritic tissue using atomic force microscopy, whereas Nesbitt et al. [25] evaluated the effect of age on the failure behavior of the meniscus through a uniaxial tensile test. In a recent study, Bansal et al. [21] investigated the changes of radial and circumferential collagen networks with maturation in terms of structure and tensile properties. Nevertheless, to the best of our knowledge, the influence of tissue development on the mechanical response of the meniscus has not yet been well-established. 

The aim of this study was therefore to evaluate the mechanical properties of the swine meniscus and to relate them to the tissue composition (glycosaminoglycan (GAG)), DNA, and collagen content) at different stages of tissue development while considering specific sites and tissue orientations. To this end, we performed tensile and compressive tests on partially and fully developed porcine menisci, and we measured the content of proteoglycans and DNA at both developmental stages. For collagen content, we referred to the values reported in the literature. Furthermore, we included a numerical analysis to explore the material constitutive equations needed to represent the mechanical response in relation to the presence of different matrix components and increased organization and regional differentiation in the structure. Only a few studies have focused on computational model for the meniscus [26,27,28]. LeRoux et al. [26] developed finite element biphasic models coupled to tensile stress–relaxation tests in order to evaluate the transversally isotropic properties of the meniscal matrix. This study underlined the need to introduce a certain degree of anisotropy in order to better describe experimental meniscal behavior. To confirm this, in the present work, we performed the numerical analysis for both tensile and compressive tests while introducing a certain degree of anisotropy. In addition, and more importantly, we investigated the contribution of the porous and viscous component based on the developmental stage of the meniscus. The outcomes of our study may help to establish the relationship between the structural organization, biomechanical properties, and tissue composition at different phases of meniscal development. This fundamental information will provide us with a better understanding of the role of each component, thus improving knowledge of the events and signals regulating the full maturation of the meniscus and leading to the better design of functional substitutes. 

## 2. Materials and Methods 

### 2.1. Sample Collection and Preparation

**Figure 1:**
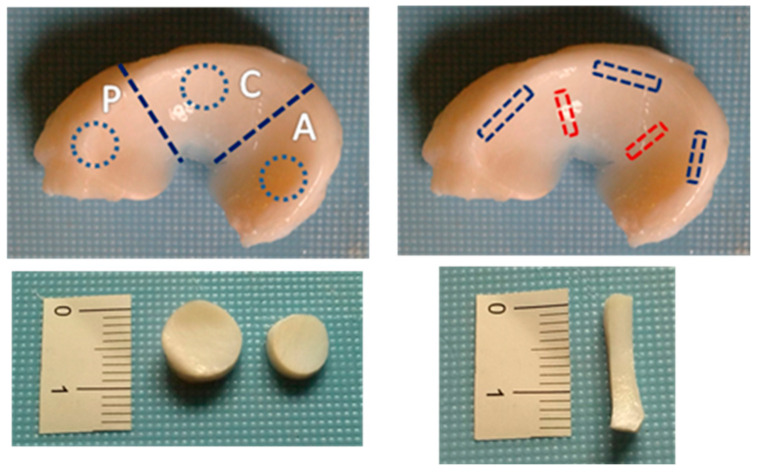
Sample preparation. Cylindrical and rectangular samples from the posterior (P), central (C), and anterior (A) regions were obtained for compression and tension tests, respectively. Stripes were obtained along the radial and circumferential directions.

Menisci were isolated from knee joints of ~7-month-old (weight 75–90 kg) and 1-month old (weight 10–12 kg) pigs obtained from a local slaughterhouse, which breeds swine Landrace x Large White, and stored at −20 °C until pending analyses. From the morphological point of view, the meniscus is fully developed in 7-month-old and partially developed in 1-month-old pigs. We then named the 7-month-old pigs FD (fully developed) and the 1-month-old pigs PD (partially developed). The swine model was chosen because of its similarities with the human meniscus, its easy availability, and its wide use in literature as a model for meniscal tissue engineering and repair [29,30,31,32]. No animal was sacrificed for the purposes of this study. Three pigs and twelve menisci for each development group were analyzed. FD menisci were cut into 2 parts along the thickness. From each meniscus sample, we obtained circular disks for compression tests and rectangular strips for tension tests (Figure 1) by using steel cutting tools. If the cylindrical samples were too irregular in thickness, we cut a thin slice from the top in order to make them flat. Each obtained specimen was then rinsed in a 0.9% saline solution and frozen at −24 °C until the time of testing. Before the test, each sample was thawed at room temperature for about 30 min in a 0.9% saline solution. Cylindrical samples (4–7 mm in diameter, 2.33 ± 0.48 SD in thickness for PD samples, and 2.63 ± 0.48 SD in thickness for SD samples) were tested in compression under an unconfined configuration. Rectangular samples (3 mm × 8 mm in length and width, 2.24 ± 0.62 SD in thickness for PD samples, and 1.83 ± 0.39 SD in thickness for SD samples) were tested in tension after measuring the thickness by a digital caliber. The locations and sectioning of all the tested samples are reported in Table 1 and Table 2, respectively. All tests were performed at room temperature using an electromagnetic testing machine (Elf3200, TA Instruments, New Castle, DE, USA) equipped with a 220 N or 22 N load cell, depending on the samples and measured load levels. 

### 2.2. Biochemical Analyses

For each experimental group (PD and FD), 6 specimens were processed (total *n* = 12). The samples were digested in papain (Sigma-Aldrich, Milan, Italy) for 16–24 h at 60 °C; the digestion solution was composed of 125 lg/mL of papain (Sigma-Aldrich) in 100 mM sodium phosphate, 10 mM sodium EDTA (Sigma-Aldrich), 10 mM cysteine hydrochloride (Sigma-Aldrich), and 5 mM EDTA adjusted to pH 6.5, and it was brought to 100 mL of solution with distilled water. The digested samples were stored at −80 °C until analyses. Aliquots of the papain digests were separately assayed for proteoglycan and DNA contents. Proteoglycan content was estimated by quantifying the amount of sulphated glycosaminoglycans using the 1,9-dimethylmethylene (DMMB) blue dye binding assay (Polysciences Inc., Washington, PA, USA) and a microplate reader (wavelength: 540 nm). The standard curve for the analysis was generated using bovine trachea chondroitin sulphate A (Sigma-Aldrich). DNA content was evaluated with the Quant-iT Picogreen dsDNA Assay Kit (Molecular Probes, Inc., Eugene, OR, USA) and a fluorescence microplate reader at standard fluorescein wavelengths (excitation: 485 nm; emission: 538 nm; cut-off: 530 nm). The standard curve for the analysis was generated using the bacteriophage lambda DNA supplied with the kit.

### 2.3. Uniaxial Tension Test

**Figure 2:**
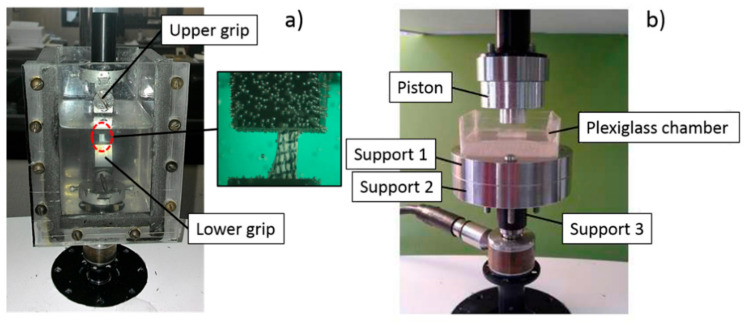
Setup for tension (**a**) and unconfined compression (**b**) tests: (**a**) the sample was clamped between two grips and kept hydrated with a saline solution in a custom-made chamber. A zoom of the sample during the tension test is shown; (**b**) the sample was placed in a Plexiglas chamber filled with a saline solution for hydration and compressed with the piston.

One side of the sample surface was marked by waterproof India ink to obtain a grid for optical strain measurements. The specimen was then mounted between the two machine jaws. A custom-made chamber filled with a 0.9% saline solution was used to keep the sample hydrated during the test (Figure 2a). The specimen was pre-loaded to 0.1 N and then subjected to a multi-ramp stress–relaxation test comprised of four increasing 4% strains at a velocity of 0.1%/s of the sample length, followed by stress–relaxation to equilibrium for 1200 s. At the end of each relaxation, images of the strained sample were acquired using a digital camera (TV Lens C−0.6X Nikon^®^) mounted on a stereomicroscope (Nikon SMZ800^®^). The Poisson coefficient was determined for FD samples as the ratio between the transversal to axial strain, and it was calculated for each tensile ramp by tracking the position of four grid points in the central portion of the sample using the NIS-Elements D camera software. The tensile relaxation elastic moduli were determined for each ramp from the equilibrium data as the ratio between the relaxation stress value and the corresponding value of strain, as determined by the machine actuator position. Besides the stress curves that showed no typical slip trend, no slippage between jaws was confirmed by image analysis. 

### 2.4. Unconfined Compression Test

The cylindrical sample was placed in a Plexiglas chamber (Figure 2b); after the contact between the sample and the piston (*Φ* = 9 mm) was achieved, the sample thickness was evaluated from the machine actuator position. The saline solution was then added to the chamber to ensure sample hydration during the test and then subjected to a multi-ramp stress–relaxation test comprised of five increasing 4% strains at a velocity of 0.1%/s of the sample thickness, followed by stress–relaxation to equilibrium for 600 s (FD menisci) or 2000 s (PD menisci). The compressive equilibrium modulus, E, was obtained for each ramp from the equilibrium data as the ratio between the values of relaxation stress and the corresponding values of the strain. For 8 FD samples, frontal images were acquired at the end of relaxation using the same optical setup described for tension tests. The Poisson coefficient under compression was then evaluated for each ramp as the ratio of the radial strain to the axial one. For the axial strain, we used the one calculated by the machine actuator, whereas the radial strain was calculated by following the lateral expansion of the sample using the NIS-Elements D camera software.

### 2.5. Statistical Analyses

The statistical analysis of the data was performed using the general linear model of the Statistic Package for Social Science (SPSS, version 27). In particular, for biochemical quantities, the differences between means in GAGs, DNA, and GAG/DNA for the two groups of FD and PD were compared by a one-way ANOVA test. Differences in the mechanical properties were compared by a two-way ANOVA test while considering several combinations of the strain level, meniscal zone (anterior, central, or posterior), load direction (circumferential or radial), and development group (FD or PD). Differences between means were considered significant at *p* < 0.05 and highly significant at *p* < 0.01.

### 2.6. Numerical Model

Computational models were implemented using the COMSOL Multiphysics commercial software (COMSOL Inc., Burlington, MA, USA) with the aim of defining the best constitutive law to represent the behavior of the meniscal tissue under a certain test configuration. The governing equations for a biphasic or viscoelastic material were used to model the behavior of meniscal samples, which were previously tested under unconfined compression and tensile tests. When using a biphasic model, we considered the solid component as either elastic or viscoelastic. The solid component was considered isotropic or transversally isotropic. The poro-elastic theory describes the interaction between the fluid domain and the solid phase in porous media. The 2D and time-dependent constitutive laws for a poro-elastic material relate total stress, strain, pore pressure, and fluid content, and they can be written as follows [33]:

(1) $$\sigma =C\varepsilon -\alpha_{B}p_{f}I$$

(2) $$p_{f}=\frac{1}{S}\left(\zeta -\alpha_{B}\varepsilon_{vol}\right)$$

(3) $$S=\frac{\varepsilon_{p}}{K_{F}}+\frac{\alpha_{B}-\varepsilon_{p}}{K_{s}}$$

where *σ* is the Cauchy stress tensor, *C* is the elasticity matrix in drained conditions, *ε* is the strain sensor, *α_B_* is the Biot–Willis coefficient, *p_f_* is the fluid pore pressure, *S* is the storage coefficient, *ζ* is the fluid content, *ε_vol_* is the volumetric strain, *K_f_* is the fluid bulk modulus (the inverse of the fluid compressibility *χ_f_*), and *K_s_* is the solid bulk modulus. The fluid equations in a poro-elastic model comes from the mass conservation, Darcy’s law, and storage model equations written as follows:

(4) $$\frac{\partial }{\partial t}\left(\rho_{f}\varepsilon_{p}\right)+\nabla \cdot \left(\rho_{f}u\right)=0$$

(5) $$\;u=\frac{-K}{\mu }\nabla p_{f}$$

(6) $$\frac{\partial }{\partial t}\left(\rho_{f}\varepsilon_{p}\right)=\rho_{f}S\frac{\partial p_{f}}{\partial t}$$

where *ρ_f_* represents fluid density, *ε_p_* is the porosity, *u* is the velocity field, *K* is the fluid permeability, and *μ* is the fluid dynamic viscosity. Therefore, if we combine the previous equations, we obtain global equations that are able to couple the solid and the fluid domains in the poro-elastic model:

(7) $$-\nabla \cdot \sigma =F_{V}$$

(8) $$\rho_{f}S\frac{\partial p_{f}}{\partial t}+\nabla \cdot \left(\rho_{f}u\right)=Q_{m}-\rho_{f}\alpha_{B}\frac{\partial }{\partial t}\varepsilon_{vol}$$

where *σ* is the total stress tensor from Equation (1), *F_V_* is the volume force term, *u* is the velocity field from Equation (5), *Q_m_* is the source term, and *∂ε_vol_*/*∂t* is the rate of change in the volumetric strain of the porous matrix. Gravity force and inertial effects were neglected in our approach.

In order to introduce viscoelastic behavior into the solid matrix, we used the generalized Maxwell model. This is a physical interpretation of the dependence of the deviatoric stress on strain history by means of the following three-branch Prony’s series relaxation function [34]:

(9) $$\Gamma \left(t\right)=E_{\infty }+\overset{3}{\underset{n=1}{\sum }}E_{n}e^{\frac{-t}{\tau_{n}}}$$

A list of the parameters used in the models is given in Table 3. Other parameters were the three components of the elastic modulus tensor along the principal directions—*E_r_*, *E_ϕ_*, and *E_z_*—and the Prony series coefficients. Elastic modulus values resulting from the analysis of the stress–relaxation tests were used as the starting values of each numerical simulation. Then, we best-fitted the experimental response σ–ε with the numerical one, choosing the most suitable constitutive law and tuning the values of the six Prony’s parameters, elastic modulus tensor components, and fluid permeability. The solid matrix is considered incompressible, whereas the porous medium is considered incompressible and isotropic: thus, the Biot–Willis coefficient and fluid compressibility were chosen equal to 1 and 10^−19^, respectively. As in the experiments, the fluid implemented in the numerical models was physiologic sodium chloride solution. The load velocity and relaxation time for the numerically-modelled compression and tension tests were the same as those of the experimental tests.

The porosity *Φ* was measured for 5 samples of FD menisci and 5 samples of PD menisci as:

(10) $$\Phi =\frac{W_{T}-W_{d}}{W_{T}}$$

where *W_T_* and *W_D_* are the total and dry sample weights, respectively, obtained after 48 h of heating at 80 °C. 

Since the porosity is about 67%, the majority of the tissue is made of water, and we therefore assumed the value of its density to be slightly higher than the one of water (1050 kg/m^3^). 

**Figure 3:**
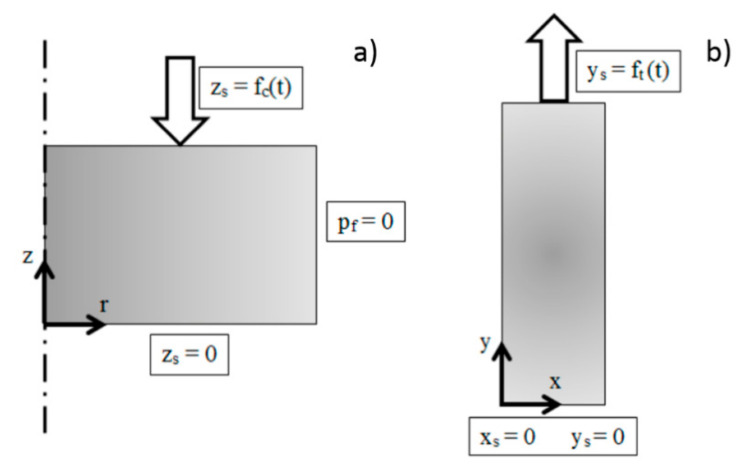
Representation of numerical model along with boundary conditions used for unconfined compression (**a**) and tension (**b**) simulations: (**a**) circular disk under unconfined compression is represented through 2D axial-symmetry. The fluid pressure (p_f_) was set to 0 at the right boundary, a multiramp compressive load (f_c_(t)) was applied to the disk in the z direction (z_s_) on the upper boundary, the movement in which was constrained at a lower boundary; (**b**) a 2D geometry was used to model the sample under tension. A multiramp tensile force (f_t_(t)) was applied to the upper boundary in the y direction (y_s_), whereas the lower boundary was constrained in both the x_s_ and y_s_ directions.

The Poisson coefficient was set to the average of the values that we measured under compression and tension tests for the FD menisci, respectively. For each specimen, we defined sample-specific geometry, different material laws (viscoelastic, poro-elastic, and poro-viscoelastic), different degrees of anisotropy (isotropic and transversally isotropic), and different boundary conditions to simulate unconfined compression or tensile loading. 2D axial-symmetry was implemented for circular disks under unconfined compression, whereas we used a 2D geometry to model the strips loaded under plane stress tension (Figure 3). Obviously, the same dimensions of the experimental tests were chosen for the model geometries for both configurations. Standard boundary conditions under unconfined compression and uniaxial tension were imposed. In order to represent the hyperelastic behavior σ–ε of the biological tissues, each of the 4 relaxation ramps was implemented with an elastic modulus corresponding to the specific level of testing strain. In this way, we could update the value of elastic modulus for each ramp starting from the experimental response of the samples. Domains were discretized with unstructured, free triangular and mapped meshes for unconfined compression and tensile simulations, respectively. The number of mesh elements ranged between 1200 and 4000, according to the type of simulation (e.g., tension, unconfined compression, and constitutive models). They were dense enough that further refinements did not significantly affect the stored solutions. We simulated five uniaxial ramps of 4% strain each. Simulations were computed using MUMPS solver and the BDF (backward differentiation formula) method, with maximum order set to five and free steps taken by the solver itself. 

## 3. Results

### 3.1. Biochemical Analyses

**Figure 4:**
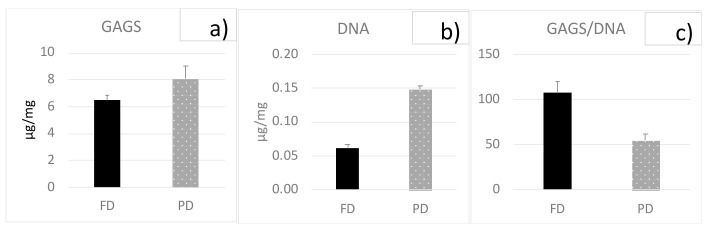
GAG and DNA contents for FD and PD samples, expressed as µg/mg of wet weight: (**a**) GAG, (**b**) DNA, and (**c**) GAG-to-DNA ratio. Bars are standard errors.

GAG deposition was similar in both the FD and PD groups, while PD samples revealed a significantly higher cellularity (*p* < 0.01). On the contrary FD samples showed a significantly higher GAG/DNA ratio (*p* < 0.01), as shown in Figure 4. Results are expressed as µg/mg of wet weight. The GAG/DNA ratio is considered an index of metabolic function.

### 3.2. Tension Tests

**Figure 5:**
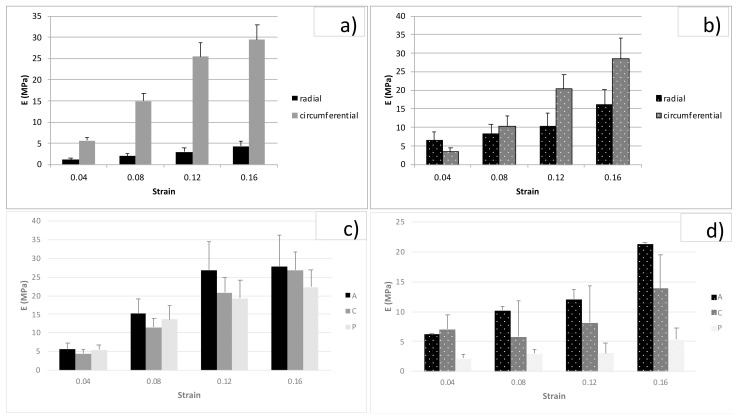
Tension tests: relaxation moduli for different strain levels, from 0.04 to 0.16, for different directions—radial and circumferential—and different zones—anterior (A), central (C), and posterior (P). (**a**) Radial and circumferential values for FD menisci; (**b**) radial and circumferential values for PD menisci; (**c**) anterior (A), central (C), and posterior (P) values for circumferential FD menisci; and (**d**) anterior (A), central (C), and posterior (P) values for circumferential PD menisci. Bars are standard errors.

**Figure 6:**
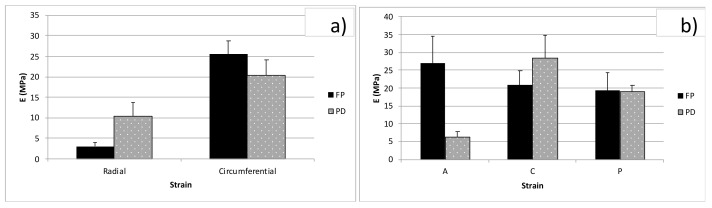
Comparison between tensile moduli for FD and PD menisci at the 0.12 strain level for (**a**) two directions—radial and circumferential—and for (**b**) different zones—A, C, and P—for circumferential samples. Bars are standard errors.

Forty-eight stripes from FD and PD menisci were tested according to the previously reported details and Table 1. Differences in E (tensile equilibrium modulus) were tested for FD and PD by two-way ANOVA tests while considering strain and direction, strain and zone, or strain and development as factors. The tension elastic modulus in the radial and circumferential direction increased with strain both for FD (Figure 5a) and PD (Figure 5b) menisci (*p* < 0.05). The circumferential direction was stiffer than the radial one for both stages of development, although differences were significant only for FD (*p* < 0.01). In particular, E in the circumferential direction was, on average, 6.82 times E in the radial direction for FD. Different zones were compared from the circumferential direction at different strain levels, as shown in Figure 5c,d for FD and PD, respectively. Differences due to strain level were significant for both FD and PD (*p* < 0.01), whereas the zone was only a significant factor for PD (*p* < 0.01). No significant difference was observed between FD and PD menisci for the circumferential direction, whereas E in the radial direction was higher for PD than FD menisci (*p* < 0.01). Though this behavior was observed for all strain levels, we only show the comparison for the 12% strain for clarity in Figure 6. The Poisson coefficient evaluated in FD menisci was 0.52 (±0.31 SD; *n* = 28) for circumferential samples and 0.28 (±0.31 SD; *n* = 4) for radial ones (n.s.).

### 3.3. Unconfined Compression

**Figure 7:**
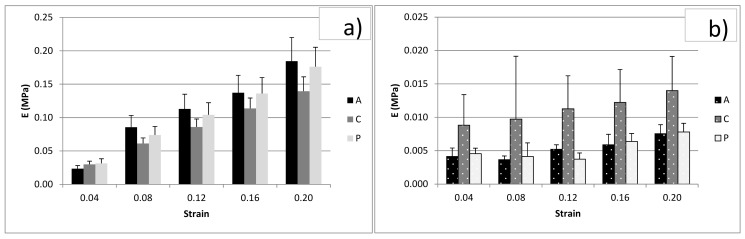
Compression tests: Moduli for different strain levels, from 0.04 to 0.20, and for different regions: anterior (A), central (C), posterior (P). (**a**) Values for FD menisci and (**b**) values for PD menisci. Bars are standard errors.

**Figure 8:**
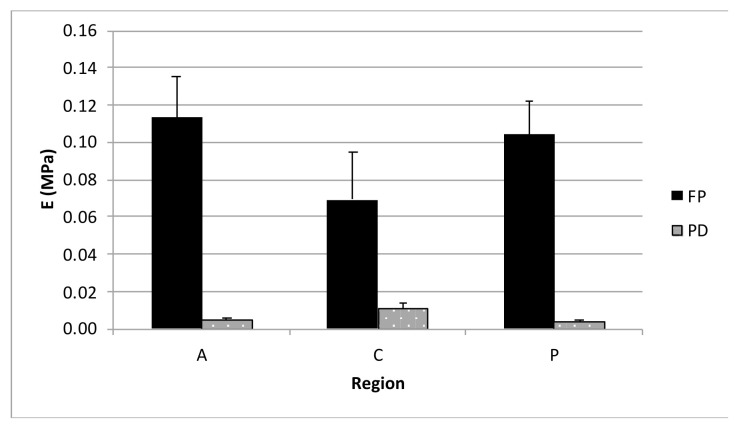
Comparison between compressive moduli for FD and PD menisci at the 0.12 strain level for three regions: anterior (A), central (C), and posterior (P). Bars are standard errors.

84 disks from FD and PD menisci were tested according to the previously reported details and Table 2. The compressive elastic modulus, E, of the FD menisci increased with strain (*p* < 0.01) for every zone (Figure 7), though differences in zones were not significant. On the contrary, the strain level for PD menisci was not significant, though differences in zones were significant (*p* < 0.01), and PD menisci in particular showed higher compressive moduli for the central region than the anterior and posterior regions. For every zone (A, C, and P) FD menisci showed higher E values than PD menisci (*p* < 0.01). The elastic moduli of FD menisci were 6–30 times higher than the E of PD menisci. As a representative behavior, we show the comparison between FD and PD for different zones at 12% strain in Figure 8. The Poisson coefficient evaluated for FD menisci was 0.08 (±0.04 SD; *n* = 8).

### 3.4. Numerical Models

**Figure 9:**
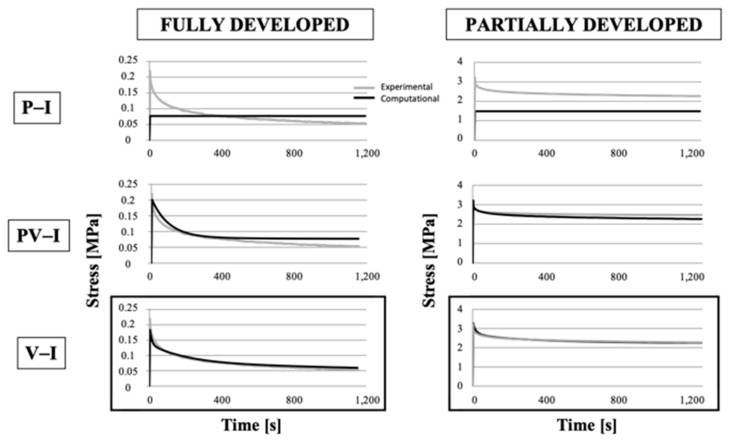
Stress–relaxation response of FD (left column) and PD (right column) meniscal rectangular samples for tension load. Comparison between experimental data and numerical results for different constitutive laws. P-I: poro-elastic isotropic; PV-I: poro-viscoelastic isotropic; V-I: viscoelastic isotropic.

Different constitutive laws were considered to simulate the experimental tests, i.e., stress–relaxation tests performed under tension or compression for both FD and PD menisci. Only one of the multi-ramp stress–relaxation curves was fitted. Tension curves were simulated by implementing poro-elastic isotropic models (P-I), poro-viscoelastic-isotropic models (PV-I), and viscoelastic-isotropic models (V-I). Considering the uniaxial load direction occurring under tension, we did not implement the transversally isotropic model for simulating tension tests. Conversely, we analyzed the model response of a pure viscoelastic material that is typically used to represent collagen fiber behavior. Stress–relaxation simulations under tensile loading for the different implemented models in comparison to experimental ones for a representative sample tested in the circumferential direction are reported in Figure 9 for FD menisci (left panels) and PD menisci (right panels). 

**Figure 10:**
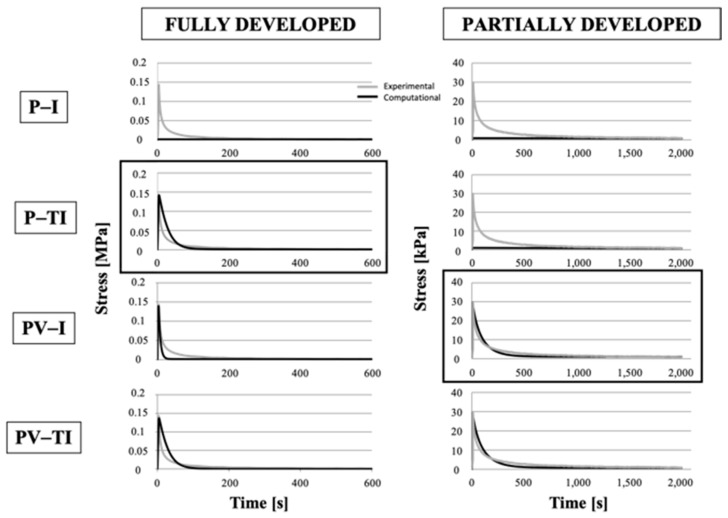
Stress–relaxation response of FD (left column) and PD (right column) meniscal discoidal samples in an unconfined compression configuration. Comparison between experimental data and numerical results for different constitutive laws. P-I: poro-elastic isotropic; P-TI: poro-elastic transversally isotropic; PV-I: poro-viscoelastic isotropic; PV-TI: poro-viscoelastic transversally isotropic.

Unconfined compression curves were simulated by implementing poro-elastic isotropic models (P-I), poro-elastic transversally isotropic models (P-TI), poro-viscoelastic isotropic models (PV-I), and poro-viscoelastic transversally isotropic models (PV-TI). Figure 10 reports the stress–relaxation response of FD and PD meniscal samples, in an unconfined compression configuration, for different material models compared to one representative experimental curve. 

The difference due to the implementation of different constitutive relations (poro-elastic, poro-viscoelastic, and viscoelastic materials) and degrees of anisotropy (isotropic and transversally isotropic materials) could be noted from the fit between experimental and numerical curves in Figure 8 and Figure 9. In both figures, the best fitting between experimental and numerical profiles is highlighted by a black square. Multi-ramp stress–relaxation curves were fit by the numerical models for all the tested samples, so Figure 8 and Figure 9 just comprise examples of what we obtained. In the case of more than one best fitting, we chose the simplest constitutive law. The numerical parameters for the best fitting models for the examples in Figure 8 and Figure 9 are reported in Table 4.

The viscoelastic model well-described the tensile response of both FD and PD menisci. However, for unconfined compression, a different behavior between FD and PD menisci was evidenced; unconfined compression curves were best fitted by the poro-elastic transversally isotropic model for the FD meniscus, but the PD menisci curves were best fitted by the poro-viscoelastic model. 

## 4. Discussion

Menisci play key roles in joint stability and load bearing. Unfortunately, they are often subjected to injuries and age-related unfavorable changes in meniscal structure. Due to its poor healing capacity, tissue engineering strategies may provide a promising solution to replace a damaged meniscus. To this end, a better understanding of the relationship between mechanical properties and structural components is needed at different developmental stages. Thus far, this correlation at different phases of development and age has been scarcely investigated [24,25]. Hence, the goal of our study was to evaluate how development and different sites and orientations influence the biomechanics of the meniscus.

Experimental tests were performed in vitro on pig menisci. As a limiting factor of this paper, the achieved findings may not be directly translated to the human meniscus, and it is difficult to replicate the effects of many years of human ageing.

The revealed mechanical properties of the swine meniscus revealed differences between samples loaded along different directions (radial or circumferential), between samples derived from pigs of different development stages, also between samples derived from different meniscal regions (anterior, central, or posterior). 

The meniscal response to tension was dependent on the loading direction and developmental stage. In particular, different values of tensile equilibrium modulus for the radial and the circumferential direction were much more marked for the FD menisci than the PD ones. Such behavior translated into changes between FD and PD menisci that were more marked in the radial direction than in the circumferential one (Figure 5). Following a study by Di Giancamillo et al. [8] that conducted the immunofluorescence localisation of collagen type 1 and 2, we observed that PD samples had a complete co-localization of collagen 1 and 2 in the inner of the middle and outer zones. Similarly, FD meniscal samples revealed a strong immunopositivity to collagen 2 in the inner zone; moreover, both collagen type 1 and 2 in the middle zone were detected, and a complete co-localization of collagen 1 and 2 in the outer zone was observed. These data were also confirmed by RT-PCR data. For these reasons, we speculate that the change in tensile mechanics between the radial and circumferential direction is due to collagen II accumulation during growth. This collagen distribution results in both higher tension moduli and a more specialized response in the circumferential direction, towards which the collagen fibers are oriented and which is the one that withstands higher loads in the FD meniscus. 

The anisotropy properties of FD menisci were confirmed by the different values of its Poisson coefficient, measured along the radial and circumferential directions, in agreement with literature data [14,16]. Our Poisson values were lower than those measured by LeRoux et al. [26] for canine menisci, although they were in agreement with other values proposed in the literature [15,35,36].

Regarding the differences between meniscal regions evidenced by the compression test results, we measured higher compressive equilibrium moduli for the anterior region of the FD meniscus compared to its central and posterior ones, in agreement with literature data [15,16,18]. For the PD meniscus, instead, higher compressive equilibrium moduli were measured for the central zones. FD menisci generally displayed much higher compressive equilibrium moduli than PD menisci. This result, combined with the higher GAG/DNA ratio measured in our FD menisci compared to PDs, can be considered an index of cell differentiation from fibroblasts in PD menisci to metabolically active chondrocytes in FD menisci. 

Values of the Poisson coefficient in compression were quite low but comparable to those measured for articular cartilage [37]. Numerical studies performed by Sweigart et al. [38] confirmed our Poisson experimental values.

The results of the numerical model simulations indicated the material constitutive laws able to represent the mechanical response of menisci of different degrees of development and under different loading conditions. The transient analysis allowed us to understand whether the characteristic behavior of the meniscus was better represented by a biphasic material, a viscoelastic material, or a combination of the two—in other words, by a poro-viscoelastic material. Furthermore, we investigated the necessity of introducing at least one level of anisotropy by comparing isotropic to transversally isotropic models. We found that the mechanical response of the meniscus could be described by different material models depending not only on the direction of load, which is not surprising because different structural components respond to different load directions, but also on the degree of development. 

Though the viscoelastic model well-described the tensile response of both FD and PD menisci, the unconfined compression of the FD meniscus was best fitted by the poro-elastic transversally isotropic model. For this tissue, poro-elasticity and transversal isotropy were found to be the major modelling features under compression, whereas the viscoelasticity was not found to play a significant role. On the contrary, viscoelasticity was found to be the major player in the response of the PD meniscus under both tension and unconfined compression. Thus, the replacement of some of the collagen I fibers with collagen II fibers occurring during growth [8] was reflected in the models by the need to include more complex material models, such as the transversal isotropy model, to describe the collagen II response under unconfined compression. Our numerical results therefore underlined the important role of the tissue components that are present in different concentrations depending on the degree of development and region and that differently respond depending on the mechanical stimulus. A limitation of our model was that the different solid matrix components (GAGs and collagen fibers) were not included, but their mechanical influence was deduced by the parameters needed to properly fit the numerical results to the experimental curves. A further development may be the characterization of meniscal samples at different degree of development by means of bi-axial tests.

## 5. Conclusions

We performed a mechanical characterization of fully developed and partially developed swine menisci by means of compression test, tension tests, and numerical analyses. The results of our investigation confirmed well known meniscal behavior, such as non-linearity, anisotropy, and non-homogeneity. Mechanical parameters were found to increase with strain, depend on the direction of load and vary among regions (anterior, central, and posterior) and directions (radial and circumferential). The novelty of our work resides in measuring changes in biomechanical properties and their link with biochemical properties occurring with development. During maturation, the GAG/DNA content ratio in tissues increases, thus augmenting compressive stiffness, whereas when collagen content and collagen type change, tissue characteristics shift from fibrous to cartilaginous, with specific mechanical responses in specialized directions. Our numerical models demonstrated the need to implement constitutive laws of increasing complexity to follow the maturation of meniscal tissue and its differentiation towards a more specialized tissue.

## Figures and Tables

**Table 1:** The number of each sample utilized for biomechanical tensile test. FD: fully developed; PD: partially developed.

	Directions	
Analysis Performed	Radial	Circumferential
Biomechanical tensile test (FD)	6 (5 anterior and 1 central)	34 (11 anterior, 14 central, and 9 posterior)
Biomechanical tensile test (PD)	11 (3 anterior, 5 central, and 3 posterior)	9 (3 anterior, 3 central, and 3 posterior)
Total samples	48	

**Table 2:** The number of each sample utilized for biomechanical unconfined compression (UC) test. FD: fully developed; PD: partially developed.

	Portions		
Analysis Performed	Anterior Horn	Central Body	Posterior Horn
Biomechanical UC (FD)	22	22	22
Biomechanical UC (PD)	6	7	5
Total samples	84		

**Table 3:** Parameters for the numerical models.

Parameter	Value	Description
vel_load_	1%∙L mm/s	Velocity of loading, referred to the reference dimension (L), i.e., thickness for compression or length for tension
t_relax_	600/1200/2000 s	Relaxation time for compression and tension tests for FD and PD menisci
ν	0.05/0.49	Poisson’s coefficient for compression and tensile tests
ρ_matrix_	1050 kg/m^3^	Drained density of solid component
ε_p_	0.675	Averaged porosity
α_B_	1	Biot–Willis coefficient
ρ	1000 kg/m^3^	Fluid density
µ	0.001 Pa∙s	Fluid dynamic viscosity
χ_f_	10^−19^ 1/Pa	Fluid compressibility

**Table 4:** Model parameter values after fitting the numerical results to the experimental data for PD and FD menisci and for unconfined compression (UC) and tension (TENSILE). P-TI: poro-elastic transversally isotropic; PV-I: poro-viscoelastic isotropic; VI: viscoelastic isotropic.

FULLY DEVELOPED			PARTIALLY DEVELOPED		
	Parameter	Value		Parameter	Value
UC: P-TI model			UC: PV-I model	K	6.26 × 10^−^^15^ m^2^
				E_z_	4.51 kPa
	K	7.67 × 10^−18^ m^2^		G_1_	136 kPa
	E_r_	15.3 MPa		G_2_	0.901 kPa
	E_ϕ_	15.3 MPa		G_3_	0.04 kPa
	E_z_	0.142 MPa		τ_1_	103 s
				τ _2_	3114 s
				τ _3_	9321 s
TENSILE: V-I model	E_z_	7.12 MPa	TENSILE: V-I model	E_z_	17.8 MPa
	G_1_	3.43 MPa		G_1_	2.33 MPa
	G_2_	3.43 MPa		G_2_	2.33 MPa
	G_3_	3.43 MPa		G_3_	2.33 MPa
	τ_1_	10 s		τ_1_	5.9 s
	τ _2_	173.3 s		τ _2_	31.9 s
	τ _3_	4200 s		τ _3_	1088 s
